# Nutritional Intervention with Antimicrobial Peptides Improves Growth Performance, Muscle Quality, Antioxidant Capacity, and Immune Function of Crucian Carp (*Carassius auratus*) Through TLR4/NF-κB Signaling Pathway

**DOI:** 10.3390/ani15172554

**Published:** 2025-08-30

**Authors:** Xiaoqing Dong, Dan Jiang, Guijuan Qu, Guiqin Wang

**Affiliations:** 1 Faculty of Animal Science and Technology, Jilin Agricultural University, Changchun 130118, China; dxq200912@163.com (X.D.); jiangdan126@163.com (D.J.); quguijuan2003@sina.cn (G.Q.); 2 Key Laboratory of Animal Production and Product Quality and Safety of the Ministry of Education, Changchun 130118, China; 3 International Joint Laboratory of Modern Agricultural Technology of the Ministry of Education, Changchun 130118, China

**Keywords:** antimicrobial peptides, crucian carp, growth performance, TLR4/NF-κB signaling pathway, immunity

## Abstract

Antimicrobial peptides (AMPs) are small-molecule polypeptides with broad-spectrum antibacterial and immunomodulatory properties. As feed additives, they have demonstrated synergistic effects in aquaculture by enhancing growth performance and maintaining host health. Its negligible drug resistance makes it an ideal additive to replace antibiotics in the “antibiotic-free breeding” system. Antimicrobial peptides were added to the basic diet of the crucian carp (*Carassius auratus*) to assess their impacts on growth, muscle quality, antioxidant capacity, immune function, and key gene expression in the TLR4/NF-κB signaling pathway. Crucian carp were fed with experimental diets containing antimicrobial peptides for 49 days, namely four treatments: L0 (0 g/kg), L1 (0.2 g/kg), L2 (0.4 g/kg), and L3 (0.6 g/kg), with three repetitions of each treatment. The findings indicated that AMPs had the potential to improve growth performance and muscle quality. The final weight, WGR, and SGR of crucian carp of group L1 significantly increased compared with groups L0 and L3 (*p* < 0.05). The condition factor of group L2 significantly increased compared with group L0 (*p* < 0.05). The FCR of groups L0, L1, and L2 was significantly reduced compared with group L3 (*p* < 0.05). The muscle redness of group L1 was significantly higher compared with groups L0, L2, and L3 (*p* < 0.05). The muscle shear force of groups L0, L1, and L2 was significantly lower compared with group L3 (*p* < 0.05). The crude protein content of groups L0, L1, and L2 showed significantly higher crude protein content than group L3 (*p* < 0.05). Conversely, the crude fat content was significantly lower in groups L1, L2, and L3 compared with group L0 (*p* < 0.05). The superoxide dismutase (SOD) activity of group L1 was significantly higher compared with groups L0, L2, and L3 (*p* < 0.05). The catalase (CAT) activity of groups L0 and L1 was significantly increased compared with groups L2 and L3 (*p* < 0.05). The malondialdehyde (MDA) content of groups L1 and L2 was significantly reduced compared with groups L0 and L3 (*p* < 0.05). The acid phosphatase (ACP) activity of groups L1 and L2 was significantly increased compared with group L0 (*p* < 0.05). The alkaline phosphatase (AKP) activity of group L1 was significantly increased compared with groups L0 and L3 (*p* < 0.05). Compared with groups L2 and L3, the lysozyme activity of group L1 was significantly increased (*p* < 0.05). The C3 content of groups L1, L2, and L3 was significantly higher compared with group L0 (*p* < 0.05). Similarly, C4 levels of groups L2 and L3 significantly exceeded group L0 (*p* < 0.05). For inflammatory cytokines, the IL-1 levels of groups L1 and L2 were significantly higher than those of group L0 (*p* < 0.05). The IL-6 and IL-12 levels of groups L0, L1, and L2 significantly increased compared with group L3 (*p* < 0.05). Compared with group L0, the levels of TNF and IFN-γ of groups L1, L2, and L3 were significantly higher (*p* < 0.05). Compared with group L0, the relative expression levels and protein expression levels of key genes TLR4, MyD88, IRAK4, TRAF6, and NF-κB of groups L1, L2, and L3 were significantly upregulated (*p* < 0.05). In conclusion, supplementation with 0.2–0.4 g/kg antimicrobial peptides promoted the growth of crucian carp, improved muscle quality, enhanced the antioxidant capacity, and boosted immunity through modulation of the TLR4/NF-κB signaling pathway.

## Simple Summary

Many farms feed large amounts of antibiotic additives long-term to prevent disease and promote fish growth, but long-term antibiotic use causes drug residues and resistance in fish, which is likely to induce disease and pollute the water environment. The results of the present study suggest that adding 0.2–0.4 g/kg of antimicrobial peptides to the diets can effectively enhance growth, improve muscle quality, increase antioxidant capacity, boost immunity, and have a positive influence on the health of crucian carp (*Carassius auratus*).

## 1. Introduction

Aquatic products are increasingly favored by people as a high-quality protein source. More and more diverse pathologies are being observed in fish farming operations because of the escalating trend towards high-density cultivation. The overuse of antibiotics and some chemicals in aquaculture to promote growth and control diseases has led to several serious problems, including the emergence of drug resistance, antibiotic residues, environmental pollution, and potential threats to public health [1]. The implementation of effective health strategies to address fish disease in aquaculture is crucial for future development. In recent years, evidence from studies has demonstrated that feed additives such as extracts from plants [2,3], organic compounds [4], and AMPs [5] can enhance fish growth and immunity. AMPs are small polypeptides with broad-spectrum antimicrobial activity. AMPs are known to possess potent activity against various pathogens, including fungi, bacteria, and viruses [6,7]. They are distributed widely in plants and animals in nature. AMPs can rapidly reach infection sites and attract immune cells to affected tissues. AMPs possess robust potential to rapidly neutralize diverse pathogens, including bacteria, fungi, and viruses. AMPs also possess the ability to neutralize endotoxins, participate in immune regulation, and induce angiogenesis in animals. These properties have positioned AMPs as one of the most promising therapeutic agents under investigation. AMPs have many advantages compared with traditional antibiotics, such as broad-spectrum antimicrobial activity, a unique antibacterial mechanism, the absence of drug residues, rapid killing action, and low resistance potential [8]. Their high-temperature stability and good water solubility enable them to withstand high temperatures during the feed granulation process. AMPs have been widely used in numerous animal feed formulations [9] and are expected to replace antibiotics and be developed into a novel aquatic feed supplement. As a feed supplement, AMPs can enhance growth and maintain the animal in a healthy state, such as promoting animal growth and enhancing the structural characteristics of the intestines [10], regulating the animal gut microbiota [11], enhancing their immune system [12,13], and promoting inhibitory effects on pathogens [14]. AMPs can also modulate the non-specific immunity of fish, promoting cytokine secretion and enhancing the immunological response of fish [15]. Adding AMPs to aquaculture can effectively improve fish health, enhance the activity of immune-related enzymes, and amplify the expression of immune-associated genes [16]. However, there are differences in effect due to the species and growth stage of fish, diet composition, living environment of fish, type of antimicrobial peptides, additive dose, and feeding duration to some extent [17]. Their dose-dependent effects on fish immunity remain underexplored.

Crucian carp (*Carassius auratus*), a predominant freshwater aquaculture species in China, faces challenges like genetic degradation and disease outbreaks under traditional farming methods. Supplementation of basal feed with AMPs can enhance growth performance and immune responses. Previous studies on common carp (*Cyprinus carpio*, average initial weight 85 g) showed no growth promotion at 0.6 g/kg AMPs [18]. Therefore, to balance dose–effect differentiation and cost control, we design a gradient dose range (0.2, 0.4, 0.6 g/kg).

To date, few studies have reported how AMPs regulate growth and immunity in crucian carp via the TLR4/NF-κB signaling pathway. Accordingly, this study aims to investigate the effects of dietary AMPs (0.2, 0.4, 0.6 g/kg) on the growth rate, muscle quality, antioxidant capacity, and immune functions in crucian carp. We hypothesize that dietary AMPs exert these regulatory effects by activating the TLR4/NF-κB signaling pathway: specifically, AMPs are predicted to upregulate the expression of key components in this pathway (e.g., TLR4, MyD88, NF-κB), which in turn enhances innate immune responses (e.g., cytokine secretion). This immune enhancement is also expected to coordinate with improved antioxidant capacity to mitigate oxidative stress-induced growth inhibition, ultimately supporting both growth and disease resistance. The study’s results are expected to provide theoretical support for sustainable aquaculture practices and practical insights into the development of eco-friendly AMP-based feed additives—addressing the industry’s need to reduce antibiotic reliance while maintaining fish productivity.

## 2. Materials and Methods

### 2.1. Diet Composition and Design

The experimental diet contained three primary protein sources: soybean meal (34%), fish meal (16%), and corn protein powder (14%), with corn oil (4%) as the principal lipid source. Other components included wheat bran (10%), flour (16.9%), calcium dihydrogen phosphate (3%), and were supplemented with 2% lysine, 5% methionine, 3% choline chloride, and 1% vitamin–mineral premix. We ground all the ingredients to pass through a 40-mesh sieve, thoroughly mixed them according to the formulation, and extruded them into pellets with a 3.0 mm diameter using an electric granulator (32-type, Mushi Machinery, Linyi City, Shandong Province, China). Pellets were dried at 60 °C to constant weight before feeding. The diet formulation and nutrient composition are shown in Table 1.

### 2.2. Feeding and Management

Feeding experiments were conducted in the aquaculture facility of Jilin Agricultural University. The experimental fish (crucian carp, *Carassius auratus*; initial weight 15.5 ± 0.5 g) were purchased from Changchun City aquatic market and acclimatized for 15 days. A total of 240 individuals were randomly allocated to 12 tanks (20 fish/tank) with water temperature maintained at 24–26 °C via thermostatic control. The fish were assigned to 4 treatment groups (*n* = 3 replicates) and administered with graded doses. Twenty fish were raised in each tank. During the experiment, the fish were fed a daily ration equivalent to 3–4% of total body weight with weekly adjustments based on growth performance. Daily feed intake and water temperature were monitored. The trial lasted 7 weeks.

### 2.3. Tissue Collection

After the experiment, 12 fish per tank were selected, and their body length/weight were measured individually. Three fish per tank were allocated to distinct analytical procedures. The fish were dissected to collect liver, pancreas, kidney, spleen, and intestinal samples for calculating the viscerosomatic index (VSI), hepatosomatic index (HSI), kidney index, and spleen index, respectively. Muscle tissues were collected for quality analysis. Blood samples were obtained via tail vein puncture from the same three fish. Blood samples were centrifuged to obtain serum for assays of antioxidant enzymes, immune-related enzymes, and non-specific immune parameters. The remaining three fish per tank were dissected, and hepatic tissues were used for gene expression analysis. The samples were flash-frozen in liquid nitrogen and stored at −80 °C until analysis.

### 2.4. Muscle Quality Analysis

Muscle color is measured using a tool called a colorimeter (HunterLab handheld chroma meter MSEZ, Hunter Associates Laboratory, Inc., Reston, VA, USA). The colorimetric measurements are expressed in CIE L*a*b* values. L* (lightness) ranges from 0 (absolute black) to 100 (pure white). a* indicates chromaticity on the red–green axis. Positive values (+a*) denote red dominance. Negative values (−a*) indicate green dominance. Zero represents neutral achromaticity. b* represents chromaticity on the yellow–blue axis. Positive values (+b*) correspond to yellow. Negative values (−b*) signify blue. Zero indicates neutrality [19]. Muscle shear force is assessed using a tenderness tester (C-LM3; initial force 5 gf; test speed 1.0 mm/s; target displacement 20 mm).

According to the method of AOAC [20], the nutritional composition of fish muscle was analyzed. Moisture content was determined by constant temperature drying at normal pressure. Crude protein was quantified using the Kjeldahl nitrogen analysis. Crude fat was measured by Soxhlet extraction with petroleum ether as solvent. Crude ash was incinerated at 550 ± 10 °C in a muffle furnace until constant weight. Calcium content was analyzed via EDTA titration. Phosphorus content was determined by the spectrophotometric molybdenum-blue method.

### 2.5. Antioxidant Capacity and Immune Indicators

The following biochemical parameters were analyzed: Catalase (CAT) and superoxide dismutase (SOD) activities were measured using assay kits (Nanjing Jiancheng Bioengineering Institute, Nanjing, China). Malondialdehyde (MDA) content was quantified via thiobarbituric acid reactive substances (TBARS) assay. Lysozyme (LZM) activity was analyzed by the turbidimetric method. The activities of alkaline phosphatase (AKP) and acid phosphatase (ACP) were analyzed using enzymatic colorimetric kits. The levels of cytokines (C3, C4, IL-1, IL-6, IL-12, TNF-α, and IFN-γ) in the serum were measured by enzyme-linked immunosorbent assay (ELISA).

### 2.6. Real-Time qPCR

The relative mRNA expression levels of target genes (*TLR4*, *MyD88*, *IRAK4*, *TRAF6*, and *NF-κB*) were quantified by real-time quantitative PCR (qPCR) using gene-specific primers designed from crucian carp sequences retrieved from the NCBI database (primer sequences listed in Table 2). The β-actin gene was used as an internal reference for normalization, and the 2^−ΔΔCt^ method was applied to calculate relative expression levels.

Total RNA was isolated from liver tissues (30–100 mg samples) using TRIzol reagent (Invitrogen, Cat# 15596-026, Carlsbad, CA, USA). The samples were homogenized in liquid nitrogen and lysed with 1 mL TRIzol reagent in 1.5 mL tubes. After adding 200 μL chloroform, the mixture was mixed until homogeneous and incubated at room temperature for 3 min. Subsequently, the mixture was centrifuged for 15 min (12,000× *g*, 4 °C). The aqueous phase was collected, and 500 μL isopropanol was added, mixed, and incubated on ice for 10 min. RNA precipitation was achieved by centrifugation (12,000× *g*, 15 min, 4 °C). The pellet was washed twice with 1 mL 75% ethanol, air-dried, and dissolved in DEPC-treated water. RNA purity was assessed by measuring A260/A280 and A260/A230 ratios (≥1.8) using a BioPhotometer spectrophotometer (Eppendorf, Hamburg, Germany). The extracted RNA was subjected to reverse transcription using the TIANScript RT KIT (Tiangen Biotechnology Co., Ltd.; Cat# KR104-02, Shanghai, China).

### 2.7. Western Blot

Cell lysate was used to isolate total protein from liver tissues (20 mg samples). Tissue was placed in EP tubes containing 200 μL cell lysate and homogenized for 1 min. The mixture was centrifuged for a duration of 10 min (12,000× *g*, 4 °C). The supernatant was retrieved and transferred to a fresh test tube. The protein concentration of the samples was subsequently assessed using the Coomassie Blue method. Proteins were separated by 10% SDS-PAGE and transferred onto PVDF membranes (0.45 μm pore size). After blocking, membranes were incubated overnight at 4 °C with primary antibodies (dilution 1:1000), then with HRP-conjugated secondary antibodies (1:3000) for 1 h at room temperature. GAPDH (Beijing Zhongshan Jinqiao Biotechnology Co., Ltd., Beijing, China.) served as the loading control. Band intensity was quantified using ImageJ software version 1.54f (NIH) after chemiluminescent detection.

### 2.8. Computation

Calculation formula of growth performance parameters:

(1) $$\mathrm{WGR}(\begin{array}{ccc} \mathrm{Weiht} & \mathrm{Gain} & \mathrm{Rate}, \end{array}\%)=\frac{(W_{t}-W_{0})\times 100}{W_{0}}\;\mathrm{SGR}(\begin{array}{ccc} \mathrm{Specific} & \mathrm{Growth} & \mathrm{Rate}, \end{array}\%)=\frac{(\mathrm{Ln}W_{t}-\mathrm{Ln}W_{0})\times 100}{t}\;\mathrm{FCR}\begin{array}{ccc} (\mathrm{Feed} & \mathrm{Conversion} & \mathrm{Rate}) \end{array}=\frac{C}{W_{t}-W_{0}}\;\begin{array}{cc} \mathrm{Condition} & \mathrm{factor} \end{array}=\frac{W_{t}}{L^{3}}\times 100\;\mathrm{Survival}(\%)=\frac{N_{t}\times 100}{N_{0}}$$

Note: *t*: time (d); *W*_0_: initial weight (g); *W_t_*: final weight (g); *C*: Feed consumption (g); *L*: body length (cm); *N*_0_: initial fish count in total; *N_t_*: final fish count in total.

Calculation formula of internal organ index:

(2) $$\begin{array}{cc} \mathrm{Visceral} & \mathrm{index}(\%) \end{array}=\frac{VW}{BW}\times 100\;\begin{array}{cc} \mathrm{Hepatopancreas} & \mathrm{index}(\%) \end{array}=\frac{HW}{BW}\times 100\;\begin{array}{cc} \mathrm{Kidney} & \mathrm{index}(\%) \end{array}=\frac{KW}{BW}\times 100\;\begin{array}{cc} \mathrm{Spleen} & \mathrm{index}(\%) \end{array}=\frac{SW}{BW}\times 100$$

Note: *VW*: visceral weight (g); *BW*: body weight (g); *HW*: hepatopancreas weight (g); *KW*: kidney weight (g); *SW*: spleen weight (g).

### 2.9. Regression Modeling

For regression modeling, a quadratic polynomial model was fitted to describe the specific growth rate:

*y* = a*x*^2^ + b*x* + c

where y represents the dependent variable; x denotes the independent variable; and a, b, c are parameters to be estimated.

Parameters were estimated via the least squares method, yielding the following fitted equation:

*y* = −0.00000131 *x*^2^ + 0.0006325 *x* + 1.3115

**Figure 1:**
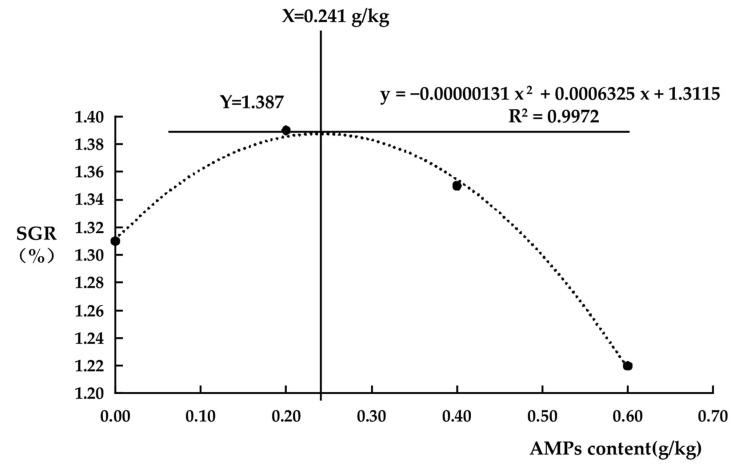
Dose–effect relationship between AMPs content and SGR (quadratic polynomial fit). X-axis: AMPs content (mg/kg); Y-axis: specific growth rate (SGR, %).

The goodness-of-fit (R^2^) of the quadratic regression model was 0.9972, indicating an excellent fit to the experimental data. The high R^2^ value suggests that AMPs content strongly explains the variation in specific growth rate (SGR), with peak efficacy occurring at approximately 0.241 g/kg (Figure 1).

## 3. Statistical Analysis

All data were presented as mean ± standard deviation. Statistical analysis was performed using one-way ANOVA, followed by post hoc multiple comparisons using LSD (Least Significant Difference) and Duncan’s tests. Significance levels were defined as follows: *p* > 0.05 (not significant, ns), *p* < 0.05 (statistically significant), and *p* < 0.001 (highly statistically significant).

## 4. Results

### 4.1. Growth Performance

The body weight of crucian carp increased nearly two-fold after the 7-week feeding trial. Growth performance and feed utilization data are presented in Table 3. The final weight, WGR, and SGR of crucian carp of group L1 showed significant increases compared to groups L0 and L3 (*p* < 0.05). Condition factor of group L2 was significantly higher than that of group L0 (*p* < 0.05). FCR of groups L0, L1, and L2 was significantly reduced compared to group L3 (*p* < 0.05). The survival rate remained 100% throughout the experiment.

The dose–effect relationship between AMPs content and SGR (quadratic polynomial fit) is displayed in Figure 1. Therefore, under the experimental conditions, the optimal dosage of growth-promoting AMPs was determined to be 0.241 g/kg.

### 4.2. Visceral Organs Index

The weights of visceral organs and the visceral index of crucian carp fed experimental diets are displayed in Table 4. Moreover, it should be noted that Table 4 did not indicate any observed differences (*p* > 0.05) in the weights of visceral organs and the visceral index among the various study groups.

### 4.3. Muscle Quality

Table 5 presents the meat color and shear force values of crucian carp fed the experimental diets. The muscle redness of group L1 was significantly higher compared to groups L0, L2, and L3 (*p* < 0.05). The muscle shear force values of groups L0, L1, and L2 were significantly lower compared to group L3 (*p* < 0.05).

The muscle composition of crucian carp is presented in Table 6. The crude protein content of groups L0, L1, and L2 was significantly higher than that of group L3 (*p* < 0.05). Conversely, the crude fat content of groups L1, L2, and L3 was significantly lower compared to group L0 (*p* < 0.05).

### 4.4. Antioxidant Abilities and Immune Function

**Figure 2:**
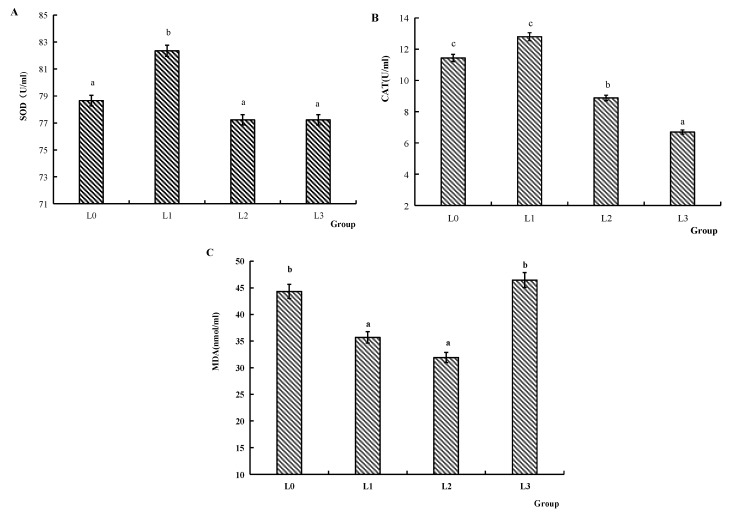
Effects of AMPs on antioxidant abilities in the serum of crucian carp. (**A**): SOD activity, (**B**): CAT activity, (**C**): MDA content. The data are presented as mean ± SD (*n* = 3). L0: control, 0 g/kg antimicrobial peptides; L1: 0.2 g/kg antimicrobial peptides; L2: 0.4 mg/kg antimicrobial peptides; L3: 0.6 g/kg antimicrobial peptides. Different letters indicate significant differences (*p* < 0.05).

Figure 2 illustrates the serum antioxidant capacity. The SOD activity of group L1 was significantly higher compared to groups L0, L2, and L3 (*p* < 0.05). The CAT activity of groups L0 and L1 was significantly increased compared to groups L2 and L3 (*p* < 0.05). The MDA content of groups L1 and L2 was significantly reduced compared to groups L0 and L3 (*p* < 0.05).

**Figure 3:**
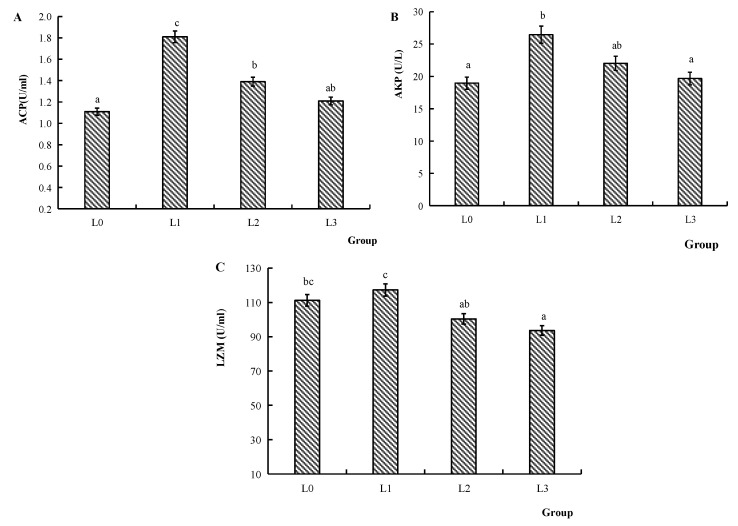
Effects of AMPs on immune-related enzyme activities in the serum of crucian carp. (**A**): ACP activity, (**B**): AKP activity, (**C**): LZM activity. The data are presented as mean ± SD (*n* = 3). L0: control, 0 g/kg antimicrobial peptides; L1: 0.2 g/kg antimicrobial peptides; L2: 0.4 g/kg antimicrobial peptides; L3: 0.6 g/kg antimicrobial peptides. Different letters indicate significant differences (*p* < 0.05).

Figure 3 illustrates the serum immune-related enzyme activities. The ACP activity of groups L1 and L2 was significantly increased compared to group L0 (*p* < 0.05). The AKP activity of group L1 was significantly increased compared to groups L0 and L3 (*p* < 0.05). Compared to groups L2 and L3, the LZM activity of group L1 was significantly increased (*p* < 0.05).

### 4.5. Immune Factors of Serum

**Figure 4:**
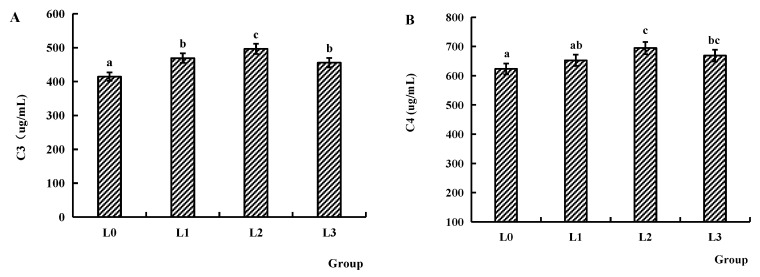

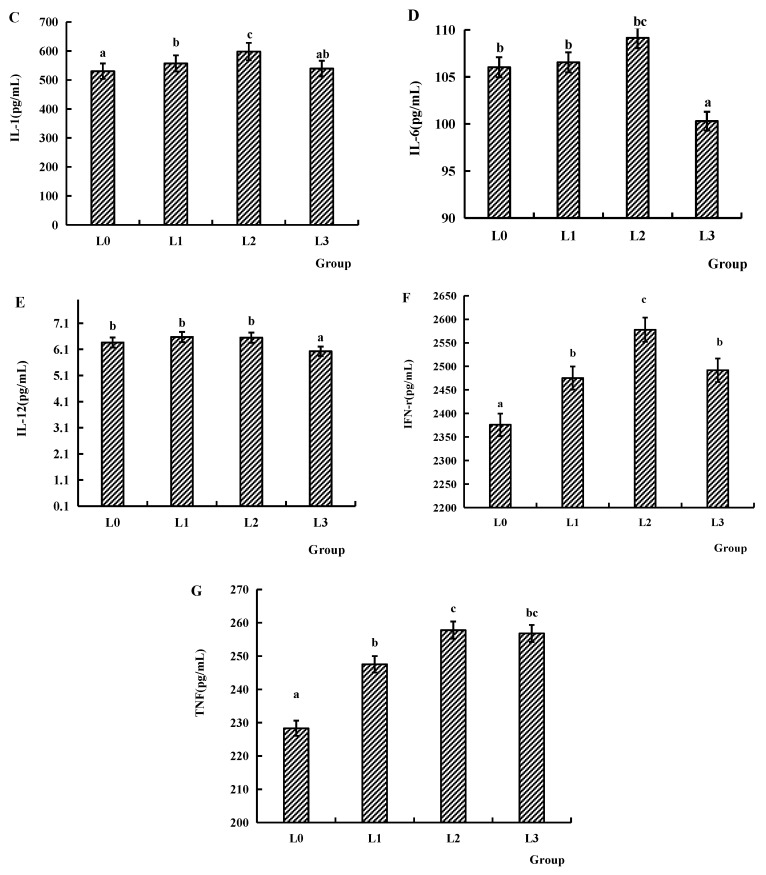
Effects of AMPs on immune factors in serum from crucian carp. (**A**): C3 content, (**B**): C4 content, (**C**): IL-1 level, (**D**): IL-6 level, (**E**): IL-12 level, (**F**): IFN-γ level, (**G**): TNF level. The data are presented as mean ± SD (*n* = 3). L0: control, 0 g/kg antimicrobial peptides; L1: 0.2 g/kg antimicrobial peptides; L2: 0.4 g/kg antimicrobial peptides; L3: 0.6 g/kg antimicrobial peptides. Different letters indicate significant differences (*p* < 0.05).

Figure 4 illustrates serum immune factor levels. The C3 content of groups L1, L2, and L3 was significantly higher compared to group L0 (*p* < 0.05). Similarly, C4 content of groups L2 and L3 was significantly higher than that of group L0 (*p* < 0.05). For inflammatory cytokines, the IL-1 level of groups L1 and L2 was significantly higher than that of group L0 (*p* < 0.05). The IL-6 and IL-12 levels of groups L0, L1, and L2 were significantly increased compared to group L3 (*p* < 0.05). The levels of TNF and IFN-γ of groups L1, L2, and L3 were significantly higher compared to group L0 (*p* < 0.05).

### 4.6. Key Gene Expression in Signaling Pathway

**Figure 5:**
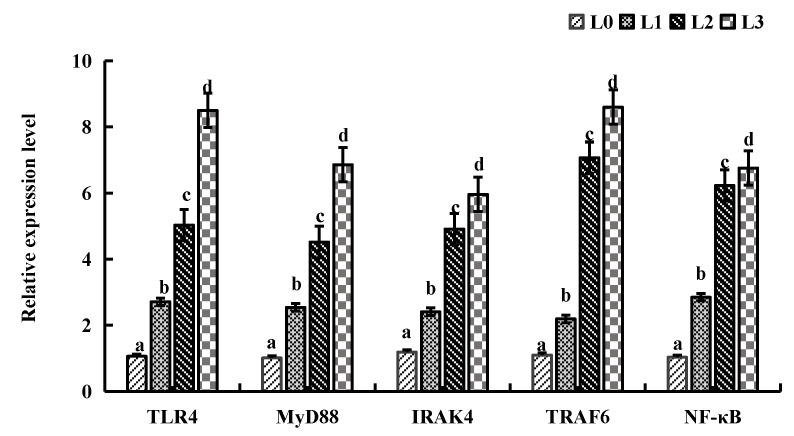
Effects of AMPs on relative expression levels of key genes in the liver tissue of crucian carp. The data are presented as mean ± SD (*n* = 3). L0: control, 0 g/kg antimicrobial peptides; L1: 0.2 g/kg antimicrobial peptides; L2: 0.4 g/kg antimicrobial peptides; L3: 0.6 g/kg antimicrobial peptides. Different letters indicate significant differences (*p* < 0.05).

Figure 5 illustrates the relative mRNA expression levels of key genes in liver tissue. Compared to group L0, the relative mRNA expression levels of key genes (*TLR4*, *MyD88*, *IRAK4*, *TRAF6*, and NF-KB) of groups L1, L2, and L3 were significantly upregulated (*p* < 0.05).

**Figure 6:**
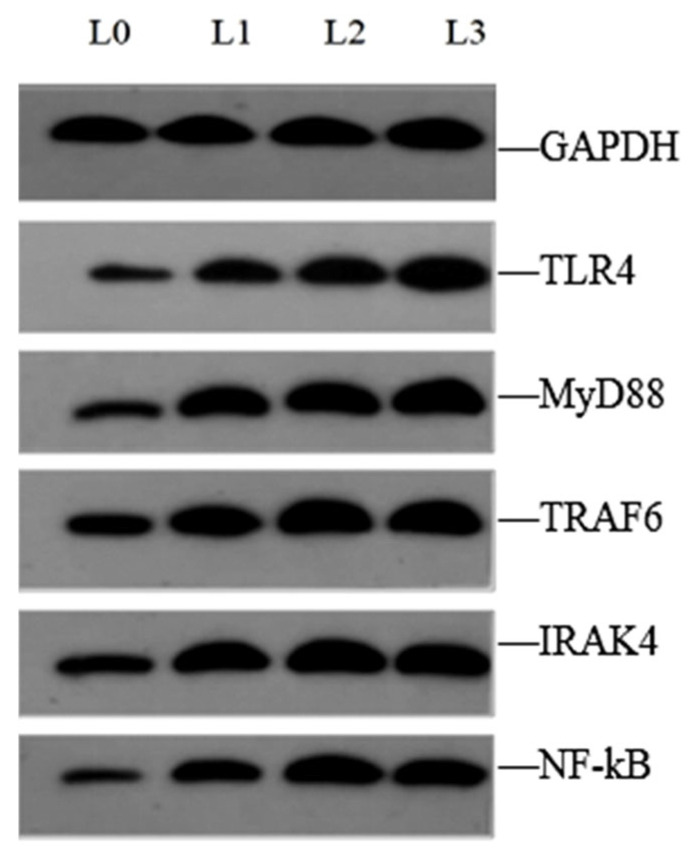
Results of TLR4, MyD88, IRAK4, TRAF6, and NF-KB Western blot liver tissue of crucian carp.

**Figure 7:**
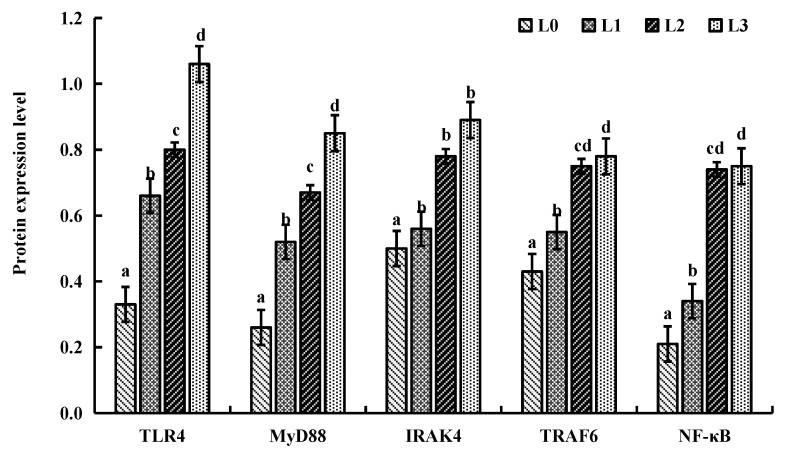
Effects of AMPs on protein expression levels of key genes in the liver tissue of crucian carp. The data are presented as mean ± SD (*n* = 3). L0: control, 0 g/kg antimicrobial peptides; L1: 0.2 g/kg antimicrobial peptides; L2: 0.4 g/kg antimicrobial peptides; L3: 0.6 g/kg antimicrobial peptides. Different letters indicate significant differences (*p* < 0.05).

Figure 6 and Figure 7 illustrate the protein expression levels of key genes in liver tissue. Compared to group L0, the protein expression levels of key genes (TLR4, MyD88, IRAK4, TRAF6 and NF-KB) of groups L1, L2, and L3 were significantly upregulated (*p* < 0.05).

**Figure 8:**
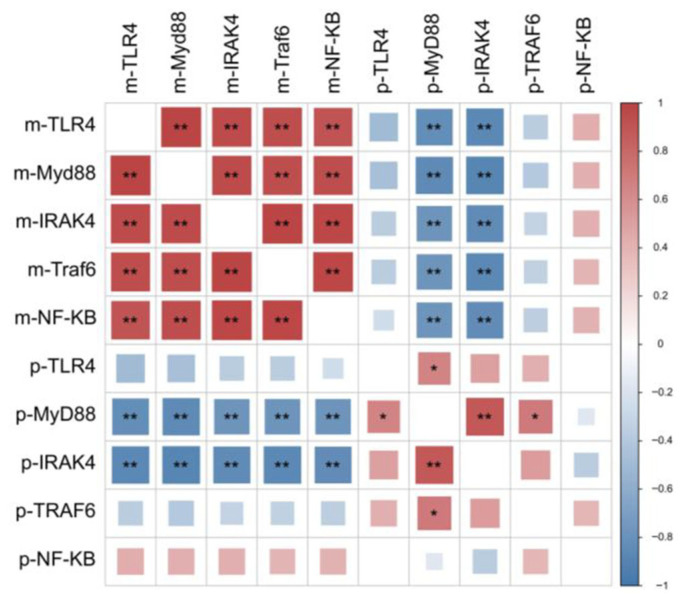
Correlation analysis of key gene expression. ** Significant correlation at 0.01 level; * Significant association at 0.05 level.

Figure 8 illustrates the correlation analysis of the relative mRNA expression levels and protein expression levels of key genes (p-TLR4, p-Myd88, p-IRAK4, p-Traf6, p-NF-κB). A significant positive correlation was observed between mRNA expression levels of these genes (*p* < 0.05). In contrast, a significant negative correlation was found between the relative mRNA expression levels and protein expression levels of these genes (*p* < 0.05).

## 5. Discussion

According to previous research, AMPs have been found to promote the growth of aquatic organisms and enhance both specific and non-specific immunity, thereby improving disease resistance [21,22]. The addition of cecropin improved the growth performance and non-specific immunity, enhanced disease resistance against Aeromonas species infection in tilapia (*Oreochromis niloticus × O.aureus*) [23]. Gyan’s studies [24] demonstrated that dietary AMPs supplementation enhanced the growth performance of *Litopeneaus vannamei*. Similarly, Jan [25] found that a dietary AMP concentration of 0.8 g/kg significantly improved the growth performance and survival rate of swamp eels. Our results indicated that when the diet was supplemented with AMPs at 0.241 g/kg, crucian carp achieved the best growth rate, whereas concentrations exceeding this level appeared to inhibit growth (compared to the control group). This study aligned with findings in aquatic species, including tilapia [5], grass carp [16], Manila clam [26], and largemouth bass [27], where dietary supplementation with AMPs at optimal doses improved growth performance. However, short-chain bioactive peptides exhibited negligible effects on growth parameters in zebrafish [22]. Notably, excessive AMP concentrations may suppress growth, likely due to dose-dependent disruption of intestinal microbiota balance. Further studies demonstrated that AMPs modulated intestinal motility and differentially influenced the colonization of pathogenic versus probiotic bacteria in fish under varied aquaculture conditions [28]. At low doses, AMPs primarily target the negatively charged cell membranes of pathogenic bacteria. However, when AMP concentrations exceed a critical threshold, they bind to all anionic membranes (including those of beneficial gut microbiota and host intestinal epithelial cells) via electrostatic interactions. This leads to membrane disruption, ultimately destabilizing the intestinal flora equilibrium and impairing nutrient absorption, which inhibits growth. These findings demonstrate that structurally distinct AMPs exert different effects in fish. Analysis of visceral organ weight and visceral somatic index revealed no significant adverse effects of AMPs on the internal organs of crucian carp, consistent with previous findings reported by Dong et al. [18].

Having established the growth-promoting effects of AMPs, we next examined their impact on muscle quality. Muscle quality can be evaluated through multiple indicators, with muscle color and tenderness being the most widely adopted parameters. Meat color serves as a crucial quality trait and represents the most visually apparent characteristic of muscle products. Notably, intramuscular fat content significantly influences meat color characteristics. Since adipocytes exhibit white coloration, increased intramuscular fat content enhances muscle brightness, consequently elevating the L* value in color measurements [29]. Fish serve as a primary source of high-quality protein, with muscle nutritional composition constituting another essential quality evaluation criterion. Muscle coloration primarily depends on three factors: tissue structure, pigment concentration, and redox status [30]. Myoglobin is the predominant muscle pigment, with its concentration and chemical state dictating ultimate color. In its native form, myoglobin appears purple, while oxygenation produces bright red oxymyoglobin—the characteristic pigment of fresh meat. Oxidation of both myoglobin and oxymyoglobin generates metmyoglobin, whose presence induces brown discoloration and contributes to muscle darkening [31]. In quality assessment, shear force is measured as a critical parameter [32], directly correlating with meat tenderness. This textural attribute is widely recognized as the principal determinant of consumer acceptability. Dietary supplementation with 0.2 g/kg AMPs significantly enhanced muscle redness (a* value) in crucian carp. The combination of low shear force and high intramuscular fat content was observed at this dosage, suggesting optimal muscle quality. However, when AMP concentration increased to 0.6 g/kg, elevated shear force values were detected, accompanied by reduced intramuscular fat content. These findings demonstrated that higher AMP concentrations adversely affect muscle tenderness parameters. Research found that dietary supplementation with additives modulated intramuscular fat deposition and enhanced porcine muscle quality [33]. Calcium plays a well-documented role in muscle tenderness through activation of endogenous calpain systems, which promote myofibrillar protein degradation [34]. Although these established mechanisms were not replicated in the present study, our findings suggested that muscle quality regulation involves complex multifactorial interactions that warrant further investigation.

Fish possess a sophisticated antioxidant defense system that plays a vital role in neutralizing oxygen-free radicals and maintaining physiological homeostasis. The antioxidant system enhances immunity, improves environmental adaptability, and reduces disease incidence. The antioxidant system of fish comprises antioxidant enzymes, including SOD and CAT, which collaboratively catalyze the neutralization of free radicals and generate non-toxic compounds. These enzymes are primary indicators of antioxidant defense, protecting cells against oxidative stress induced by excessive reactive oxygen species (ROS) [35]. MDA is a key byproduct of lipid peroxidation [36]. This study demonstrated that dietary supplementation with 0.2 g/kg AMPs increased SOD and CAT activities, reduced serum MDA content, thereby enhancing the antioxidant capacity of crucian carp. These findings are consistent with previous studies in zebrafish [22], grass carp [37], and grouper [38], suggesting that AMPs improve antioxidant responses and may mitigate immune stress.

Aquatic-dwelling fish have evolved unique immune systems to counteract environmental pathogens [39,40]. Their defense mechanisms predominantly rely on non-specific and specific immunity. Furthermore, there is a complicated cytokine network in fish for the regulation and activation of their immune system, and for producing appropriate protective responses to various pathogens [41]. Non-specific immune factors comprise lysozyme, complement proteins, and cytokines. Lysozyme is a bacteriolytic enzyme that can regulate hormonal activation of both complement systems and phagocytes, with its levels increasing post-immune stimulation [42]. ACP fulfills a critical function in the immune system by participating in intracellular degradation and metabolic processes. Its activity is often used as an indicator to evaluate cellular immune function and the health status of the organism. AKP plays an important role in calcium–phosphorus balance, metabolic regulation, and cell membrane repair. Its activity fluctuations can reflect bone development, liver function, and intestinal health [43]. In this experiment, supplementation with 0.2 g/kg AMPs significantly elevated the ACP, AKP, and lysozyme activities in the serum of crucian carp. These findings on common carp [15] and eel [44] exhibit congruent patterns with the present experimental data. AKP activity exhibited a decline when supplemented with 0.6 g/kg AMPs. This may be due to the excessive AMPs, which may impose a burden on the liver and intestines, resulting in reduced AKP activity.

The complement system critically contributes to inflammation induction, pathogen opsonization and lysis, as well as augmentation of antibody-mediated humoral immunity [45,46]. Meng et al. [47] demonstrated that elevated serum C3 levels enhance resistance to secondary infections in grass carp. Additionally, they identified C4 as a pivotal modulator of antibacterial immune responses in this species. He considered that the serum C3 quantification could serve as a diagnostic biomarker for disease status assessment. Our experimental results demonstrated that dietary supplementation with AMPs at 0.2, 0.4, and 0.6 g/kg significantly increased serum complement component C3 content in crucian carp, and doses of 0.4 and 0.6 g/kg additionally elevated serum C4 content. This is consistent with the experimental results that antimicrobial peptides induced by Hermetia illucens can increase the complement C3 content in the serum of common carp [48].

In addition to changes in serum complement components, which can reflect the state of immune activation in the organism, the regulatory roles of other key immune cytokine factors in the immune response are equally important. Cytokines, which exist in an inactivated or low-secretion state under normal conditions, are upregulated upon immunological stimulation and exert immunoregulatory functions by binding to specific receptors on the cell surface [48]. In fish, the major cytokine families include interferons (IFNs), interleukins (ILs), and tumor necrosis factors (TNFs), etc. Among these cytokines, interleukins serve as pivotal regulators of fish immune responses, regulating the proliferation, differentiation, maturation, and activation of immune cells [49]. IL-6 is a critically important cytokine that promotes the activation of T cells, B cells, and macrophages. IL-6 also upregulates the expression of proinflammatory factors. Notably, IL-6 exerts a synergistic effect with interleukin-1 (IL-1) and tumor necrosis factor (TNF) to regulate the immune response [50]. IL-12 enhances Th1 immune response and promotes IFN-γ production. In inflammatory conditions and Th1 responses, IFN-γ and IL-12 form a positive feedback mechanism to induce the production of various cytokines (e.g., IL-2 and IL-10) and further modulate the immune response [51]. TNF is a cytokine family that includes TNF-α and TNF-β, and is primarily secreted by lymphocytes and macrophages. Specifically, TNF-α is synthesized by mononuclear macrophages and exhibits dual biological functions: cytotoxic activity and regulatory effects on leukocyte migration, proliferation, differentiation, and apoptosis [52]. Interferons (IFNs) constitute a family of pleiotropic cytokines produced by nucleated cells, with their biosynthesis requiring active cellular metabolism, including RNA transcription and protein translation [53]. In mammals, type II IFNs encode only one gene (IFN-γ), while type II IFNs in fish have two members, which are IFN-γ and IFN-γ rel [54]. IFN-γ in mammals is generally considered a cytokine that regulates both innate and adaptive immune responses, particularly exerting antimicrobial effects against intracellular infections. IFN-γ in fish shares similar functions with its mammalian counterpart: for example, IFN-γ derived from rainbow trout and goldfish can enhance the respiratory burst and nitric oxide (NO) production of macrophages, as well as promote their phagocytic activity [55,56]. Studies have found that IFN-γ of grass carp is also involved in regulating the interaction of *Edwardsiella piscicida* with NOD1 or autophagosomes, thus enhancing cellular bacterial clearance [57]. Our experimental results demonstrated a dose-dependent immunomodulatory effect of dietary AMPs in crucian carp. Supplementation of 0.2–0.6 g/kg AMPs per diet significantly elevated serum IL-1 levels, while the highest dose (0.6 g/kg) significantly reduced serum interleukin-6 (IL-6) and interleukin-12 (IL-12) levels. Importantly, all tested AMP concentrations (0.2, 0.4, and 0.6 g/kg) effectively enhanced both IFN-γ and TNF-α production in serum, suggesting a potent immunostimulatory capacity of AMPs. These findings align with observations in quails, in which dietary supplementation with 0.2–0.4% AMPs similarly upregulated serum IFN-γ levels [58]. As an exogenous immunomodulator, AMPs may first trigger the immune response by activating the innate immune system of fish. They activate the adaptive immune system through the cytokines secreted by innate immune cells. Subsequently, AMPs regulate the protein secretion of cytokines in immune cells, leading to a significant increase in the levels of target cytokines (e.g., IL-1, IFN-γ, TNF-α) in the serum.

The innate immune system of teleost fish constitutes the first line of defense against pathogenic invasion and environmental stressors, which is mediated by complex networks of immune-related genes and signaling cascades [59]. As key components of this system, immune-related genes, such as toll-like receptor 4 (TLR4) in rainbow trout (*Oncorhynchus mykiss*), play a crucial role in the fish immune response [60]. Toll-like receptors (TLRs) recognize distinct microbial components to initiate the innate and adaptive immune responses. TLR activation culminates in the expression of appropriate proinflammatory and immunomodulatory factors to meet pathogenic challenges. The transcription factor NF-kB is the master regulator of all TLR-induced responses, and its activation is the pivotal event in TLR-mediated activation of the innate immune response [61]. NF-kB acts as a central regulator of the proinflammatory signaling pathway to trigger the expression of cytokines, chemokines, and adhesion molecules and modulate innate immune function [62,63].

Our study reveals that dietary supplementation with 0.2–0.6 g/kg diet of AMPs in crucian carp elicits a dual upregulation of mRNA and protein expression levels of key components in the TLR4 signaling cascade—including TLR4, IRAK4, MyD88, TRAF6, and NF-κB—in hepatic tissues. Notably, this finding provides novel evidence that AMPs can actively engage the TLR4/NF-κB pathway in teleosts. Furthermore, our data support the notion that AMPs may enhance the release of proinflammatory cytokines (TNF-α and IL-1β) through activation of this pathway, thereby uncovering a previously underappreciated immunomodulatory mechanism of dietary AMPs in crucian carp. The TLR4/NF-κB activation observed in our study aligns with conserved innate immune regulatory mechanisms across teleost species. Pan et al. [64] documented significant upregulation of MyD88, TLR4, NF-κB, and TNF-α in hepcidin-related antibacterial studies, reinforcing that AMPs—including hepcidin (an AMP family member) consistently target core components of this signaling axis. Zhang et al. [65] identified that Trachinotus ovatus hepcidins (TroHepc1/2) activate immune cells and cytokines. Our study delineates that teleost AMPs exert these effects specifically through TLR4/NF-κB engagement. We demonstrate dual transcriptional and translational regulation, a detail rarely reported in fish AMP studies but critical for confirming functional pathway activation.

**Figure 9:**
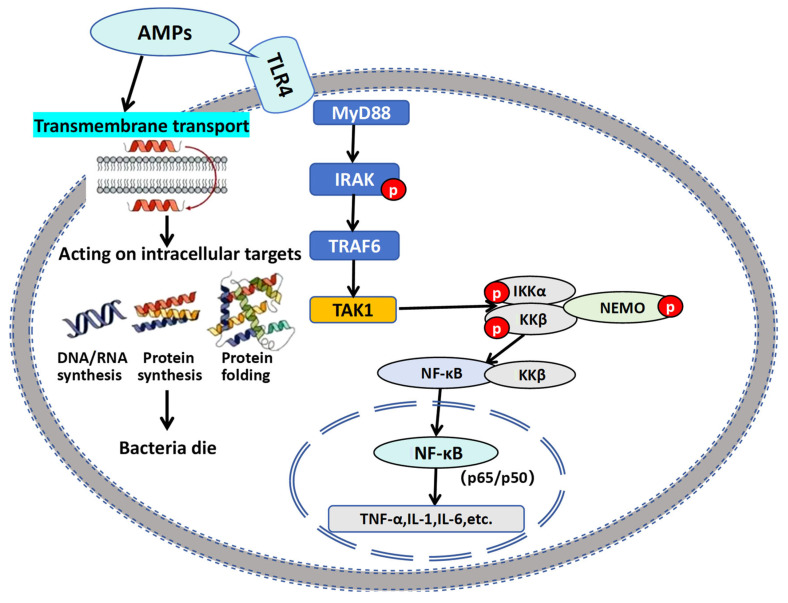
Pattern recognition receptors TLR4 activate the immune regulatory pathway of NF-κB.

To further clarify the molecular basis of how AMPs engage the TLR4/NF-κB pathway, we integrated our findings with established signaling frameworks in aquatic species. Collectively, these studies establish that AMPs serve as crucial immunomodulators by two core mechanisms: (1) regulating innate immune responses through pattern recognition receptor (PRR) pathways; (2) orchestrating cytokine networks. AMPs bind to the TLR4/MD-2/CD14 complex on cell membranes via their positively charged domains, inducing conformational changes and dimerization of TLR4. The intracellular TIR domain of TLR4 then recruits the MyD88 adapter protein, which subsequently activates the IRAK kinase family. Through the TRAF6-TAK1-IKK cascade, this process triggers phosphorylation and ubiquitin-mediated degradation of IκBα, ultimately promoting nuclear translocation of NF-κB (p65/p50) dimers. Once in the nucleus, NF-κB binds to κB sites in the promoters of target genes, systematically regulating downstream immune responses (Figure 9).

This nuclear translocation of NF-κB drives a cascade of immune effects that amplify and refine the host’s defense response. Elevated TNF-α further strengthens the TLR4/NF-κB signaling pathway via positive feedback mechanisms, enhancing endothelial adhesion molecule expression and neutrophil/macrophage recruitment. Additionally, NF-κB induces pro-IL-1β transcription, which is cleaved by caspase-1 into mature IL-1β. This activates the NLRP3 inflammatory body and enhances macrophage bactericidal activity. Cellular immune regulation drives NF-κB-dependent IL-12 expression to promote Th1 cell differentiation. Simultaneously, the TLR4-TRIF-IRF3 signaling pathway induces interferon production (IFN-α/β), which stimulates T/NK cells to secrete IFN-γ. This mechanism ultimately enhances macrophage M1 polarization and antimicrobial capabilities. Notably, our observations also highlight species-specific nuances that demand attention in practical applications: compared with mammals, the TLR4 of crucian carp exhibits higher sensitivity to AMPs, while its complement system activation threshold remains lower. Prolonged or excessive AMP exposure may therefore lead to excessive complement consumption and cytokine storms, potentially causing tissue damage. This underscores the importance of strictly controlling AMP additive doses in aquaculture practices—balancing effective immune activation with the avoidance of harmful inflammatory overactivation. In summary, our work not only validates a conserved AMP-TLR4/NF-κB regulatory axis in teleosts but also provides actionable insights for optimizing AMP-based immunostimulants in sustainable cyprinid farming.

The demonstrated nutritional and immunoregulatory benefits position AMPs as promising functional additives for sustainable aquaculture practices. AMPs play a crucial role in defending against pathogenic microorganisms and are an essential component of innate immunity [66]. As small-molecule bioactive peptides, AMPs exhibit broader-spectrum inhibitory or bactericidal activity against pathogens compared to chemical drugs such as antibiotics [67]. They also demonstrate lower cytotoxicity towards non-target organisms (e.g., farmed fish) [68]. Importantly, AMPs have a low propensity to induce pathogen resistance, and are less likely to accumulate in the environment—thus minimizing ecological toxicity and rendering them relatively environmentally friendly [65]. Consequently, antimicrobial peptides have emerged as a more ecologically sustainable alternative to antibiotics. However, many challenges in high production costs and stability remain to be addressed in practical aquaculture [69]. Addressing these bottlenecks, such as developing microbial fermentation technologies or designing microencapsulation AMPs to enhance stability, will be pivotal for antibiotic-free aquaculture.

## 6. Conclusions

Overall, dietary supplementation with AMPs at 0.2–0.4 g/kg of diet optimally enhances growth performance and muscle quality in crucian carp, while exhibiting no adverse effects on visceral organs. The findings reveal three novel mechanisms: first, AMPs uniquely synchronize growth promotion (via improving nutrient absorption efficiency) and immune enhancement—a contrast to conventional additives that often prioritize single outcomes. Second, the unchanged hepatosomatic index confirms visceral organ safety at the identified effective dose range (0.2–0.4 g/kg). Third, AMPs elevate systemic antioxidant capacity (via increasing SOD activity) while activating the TLR4/NF-κB pathway and inducing secretion of proinflammatory cytokines (IL-6, TNF-α). This dual modulation indicates that AMPs mitigate immunosuppression induced by oxidative stress. However, further investigations are warranted to elucidate the underlying mechanisms of these observations. Notably, the identified optimal dose range (0.2–0.4 g/kg of diet) balances immune stimulation while avoiding the NF-κB overactivation observed in other teleost species at higher AMP doses. This finding provides practical guidance for the application of AMPs as feed additives in freshwater cyprinid farming.

## Figures and Tables

**Table 1:** Diet formulation and nutrient composition (g/kg).

Ingredients	L0	L1	L2	L3
Antimicrobial peptides	0	0.2	0.4	0.6
Fish meal	160	160	160	160
Soybean meal	340	340	340	340
Corn oil	40	40	40	40
Wheat bran	100	100	100	100
flour	169	169	169	169
Corn protein powder	140	140	140	140
Ca (H_2_PO_4_) _2_	30	30	30	30
Premix ^a^	10	10	10	10
Lys	2	2	2	2
Met	5	5	5	5
Choline chloride (50%)	3	3	3	3
VC (35%)	1	1	1	1
Proximate composition (%)				
Dry matter	92.69	92.59	92.23	92,11
Crude protein	32.45	32.03	32.09	32.16
Crude lipid	6.65	6.74	6.85	6.57
Ash	8.77	8.71	8.73	8.86
Calcium	1.27	1.24	1.26	1.28
Total phosphorus	1.50	1.51	1.49	1.51
Energy (MJ/kg)	18.57	18.63	18.57	18.58

Note: ^a^ Composite premix additive includes vitamins and minerals. Vitamin A (IU/kg) 360,000; vitamin D_3_ (IU/kg) 12,000; vitamin E (mg/kg) 2000; vitamin K_3_ (mg/kg) 500; vitamin B_1_ (mg/kg) 500; vitamin B_2_ (mg/kg) 700; vitamin B_6_ (mg/kg) 600; vitamin B_12_ (mg/kg) 2; calcium pantothenate (mg/kg) 170; niacin (mg/kg) 3000; folic acid (mg/kg) 170; biotin (mg/kg) 5; VC (mg/kg) 17140; inositol (mg/kg) 9000; magnesium (mg/kg) 15,000; iron (mg/kg) 12,000; zinc (Mg/kg) 6000; manganese (mg/kg) 3000; copper (mg/kg) 400; cobalt (mg/kg) 50; selenium (mg/kg) 10; iodine (mg/kg) 100.

**Table 2:** Primers sequences.

Gene	ID	Primer Orientation	Size	Primer Sequence
TLR4	132145260	Forward	141	GCGAGTCAGAACCTCAGCCAATG
TLR4	132145260	Reverse	141	TCCCAAACCCTTGCTCCCTCTC
MyD88	403145	Forward	85	AGGTGCAAGAGGATGGTGGTAGTC
MyD88	403145	Reverse	85	GGCTGAGTGCGAACTTGGTCTG
IRAK4	393132	Forward	88	GGCTTTGGCGTCGTCTTCAGAG
IRAK4	393132	Reverse	88	CTTCAAGTGAGCTGTCGTCCATAGG
TRAF6	554561	Forward	110	TACGAGTGCCCTATCTGTCTGATGG
TRAF6	554561	Reverse	110	TTCTGCCCTGTGTCCCTGATGG
NF-κB	122880105	Forward	99	AGGCGGTAAGGAAGTTGCGAATG
NF-κB	122880105	Reverse	99	CAGGCTGGTCTGAAGTGTGGTTC

**Table 3:** Effects of antimicrobial peptides on growth performance of crucian carp.

Parameters	Group				
	L0	L1	L2	L3	*p*-Value
Initial weight (g)	15.50 ± 0.50	15.50 ± 0.50	15.50 ± 0.50	15.50 ± 0.50	-
Final weight (g)	29.42 ± 0.32 ^b^	30.63 ± 0.54 ^c^	29.99 ± 0.53 ^bc^	28.22 ± 0.23 ^a^	0.001
WGR (%)	89.83 ± 2.07 ^b^	97.63 ± 3.50 ^c^	93.46 ± 3.45 ^bc^	82.04 ± 1.49 ^a^	0.001
SGR (%)	1.31 ± 0.03 ^b^	1.39 ± 0.04 ^c^	1.35 ± 0.04 ^bc^	1.22 ± 0.02 ^a^	0.001
Condition factor	2.59 ± 0.08 ^a^	2.70 ± 0.01 ^ab^	2.82 ± 0.11 ^b^	2.71 ± 0.06 ^ab^	0.038
FCR	1.32 ± 0.01 ^a^	1.29 ± 0.01 ^a^	1.33 ± 0.01 ^a^	1.42 ± 0.05 ^b^	0.002
Survival (%)	100	100	100	100	-

L0: control, 0 g/kg antimicrobial peptides; L1: 0.2 g/kg antimicrobial peptides; L2: 0.4 g/kg antimicrobial peptides; L3: 0.6 g/kg antimicrobial peptides. The shoulder letter was marked differently for two groups, indicating a significant distinction (*p* < 0.05).

**Table 4:** Effects of antimicrobial peptides on visceral organ weight and visceral index in crucian carp.

Parameters	Group				
	L0	L1	L2	L3	*p*-Value
Visceral weight (g)	3.09 ± 0.16	2.79 ± 0.57	2.45 ± 0.31	2.73 ± 0.78	0.550
Hepatopancreas weight (g)	0.66 ± 0.05	0.68 ± 0.09	0.54 ± 0.08	0.71 ± 0.23	0.467
Spleen weight (g)	0.08 ± 0.02	0.06 ± 0.01	0.06 ± 0.005	0.08 ± 0.02	0.214
Kidney weight (g)	0.15 ± 0.01	0.13 ± 0.04	0.11 ± 0.01	0.18 ± 0.04	0.178
Intestines weight (g)	0.99 ± 0.19	0.72 ± 0.13	0.73 ± 0.17	0.93 ± 0.28	0.298
Intestines length (cm)	24.14 ± 0.42	22.47 ± 3.07	20.85 ± 0.99	23.02 ± 4.18	0.527
Visceral index (%)	10.27 ± 0.71	9.47 ± 1.88	8.32 ± 0.80	9.65 ± 2.70	0.600
Hepatopancreas index (%)	2.20 ± 0.12	2.31 ± 0.29	1.84 ± 0.23	2.54 ± 0.80	0.349
Spleen index (%)	0.28 ± 0.07	0.21 ± 0.36	0.21 ± 0.01	0.28 ± 0.07	0.259
Kidney index (%)	0.50 ± 0.01	0.46 ± 0.12	0.38 ± 0.03	0.62 ± 0.16	0.094

L0: control, 0 g/kg antimicrobial peptides; L1: 0.2 g/kg antimicrobial peptides; L2: 0.4 g/kg antimicrobial peptides; L3: 0.6 g/kg antimicrobial peptides.

**Table 5:** Effects of AMPs on meat color and shear stress of crucian carp (fresh sample).

Parameters	Group				
	L0	L1	L2	L3	*p*-Value
Lightness (L*)	44.81 ± 3.54	42.61 ± 0.67	42.54 ± 1.06	44.99 ± 0.82	0.291
Redness (a*)	3.35 ± 1.01 ^a^	5.09 ± 0.36 ^b^	3.44 ± 0.22 ^a^	2.23 ± 0.68 ^a^	0.004
Yellowness (b*)	10.22 ± 0.96	10.10 ± 0.56	10.11 ± 0.76	9.80 ± 0.18	0.890
Shear force (N)	9.92 ± 0.93 ^a^	10.32 ± 0.35 ^a^	9.93 ± 0.94 ^a^	12.70 ± 0.45 ^b^	0.004

L0: control, 0 g/kg antimicrobial peptides; L1: 0.2 g/kg antimicrobial peptides; L2: 0.4 g/kg antimicrobial peptides; L3: 0.6 g/kg antimicrobial peptides. L* represents the lightness of the muscle, a* represents the redness of the muscle, and b* rep-resents the yellowness of the muscle. The shoulder letter was marked differently for two groups, indicating a significant distinction (*p* < 0.05).

**Table 6:** Effects of AMPs on muscle composition of crucian carp (dry basis).

Parameters	Group				
	L0	L1	L2	L3	*p*-Value
Water (%)	9.25 ± 1.09	8.28 ± 0.83	8.51 ± 1.47	8.33 ± 0.40	0.651
Crude protein (%)	64.63 ± 1.50 ^b^	65.51 ± 1.87 ^b^	66.24 ± 1.40 ^b^	56.77 ± 1.95 ^a^	<0.001
Crude fat (%)	8.33 ± 0.56 ^d^	6.92 ± 0.20 ^c^	5.51 ± 0.34 ^b^	3.49 ± 0.12 ^a^	<0.001
Crude ash (%)	7.99 ± 1.26	8.49 ± 0.39	7.84 ± 0.18	8.58 ± 0.63	0.557
Ca (%)	0.32 ± 0.06	0.34 ± 0.05	0.30 ± 0.05	0.28 ± 0.04	0.499
P (%)	0.40 ± 0.03	0.41 ± 0.02	0.39 ± 0.03	0.38 ± 0.02	0.401

L0: control, 0 g/kg antimicrobial peptides; L1: 0.2 g/kg antimicrobial peptides; L2: 0.4 g/kg antimicrobial peptides; L3: 0.6 g/kg antimicrobial peptides. The shoulder letter was marked differently for two groups, indicating a significant distinction (*p* < 0.05).

## Data Availability

The original contributions presented in this study are included in the article. Further inquiries can be directed to the corresponding author.
